# A persistent lack of international representation on editorial boards in environmental biology

**DOI:** 10.1371/journal.pbio.2002760

**Published:** 2017-12-12

**Authors:** Johanna Espin, Sebastian Palmas, Farah Carrasco-Rueda, Kristina Riemer, Pablo E. Allen, Nathan Berkebile, Kirsten A. Hecht, Kay Kastner-Wilcox, Mauricio M. Núñez-Regueiro, Candice Prince, Constanza Rios, Erica Ross, Bhagatveer Sangha, Tia Tyler, Judit Ungvari-Martin, Mariana Villegas, Tara T. Cataldo, Emilio M. Bruna

**Affiliations:** 1 Department of Sociology and Criminology & Law, University of Florida, Gainesville, Florida, United States of America; 2 Tropical Conservation and Development Program, Center for Latin American Studies, University of Florida, Gainesville, Florida, United States of America; 3 School of Forest Resources and Conservation, University of Florida, Gainesville, Florida, United States of America; 4 School of Natural Resources and Environment, University of Florida, Gainesville, Florida, United States of America; 5 Department of Wildlife Ecology & Conservation, University of Florida, Gainesville, Florida, United States of America; 6 Entomology and Nematology Department, University of Florida, Gainesville, Florida, United States of America; 7 Florida Museum of Natural History, University of Florida, Gainesville, Florida, United States of America; 8 Soil and Water Sciences Department, University of Florida, Gainesville, Florida, United States of America; 9 Department of Environmental Horticulture, University of Florida, Gainesville, Florida, United States of America; 10 Horticultural Sciences Department, University of Florida, Gainesville, Florida, United States of America; 11 Department of Biology, University of Florida, Gainesville, Florida, United States of America; 12 Martson Science Library, University of Florida, Gainesville, Florida, United States of America

## Abstract

The scholars comprising journal editorial boards play a critical role in defining the trajectory of knowledge in their field. Nevertheless, studies of editorial board composition remain rare, especially those focusing on journals publishing research in the increasingly globalized fields of science, technology, engineering, and math (STEM). Using metrics for quantifying the diversity of ecological communities, we quantified international representation on the 1985–2014 editorial boards of 24 environmental biology journals. Over the course of 3 decades, there were 3,827 unique scientists based in 70 countries who served as editors. The size of the editorial community increased over time—the number of editors serving in 2014 was 4-fold greater than in 1985—as did the number of countries in which editors were based. Nevertheless, editors based outside the “Global North” (the group of economically developed countries with high per capita gross domestic product [GDP] that collectively concentrate most global wealth) were extremely rare. Furthermore, 67.18% of all editors were based in either the United States or the United Kingdom. Consequently, geographic diversity—already low in 1985—remained unchanged through 2014. We argue that this limited geographic diversity can detrimentally affect the creativity of scholarship published in journals, the progress and direction of research, the composition of the STEM workforce, and the development of science in Latin America, Africa, the Middle East, and much of Asia (i.e., the “Global South”).

## Introduction

There are currently over 28,000 peer-reviewed academic journals [1], and the scholars who serve on the editorial boards of these journals play a major role in defining the trajectory and boundaries of knowledge in their disciplines [2]. This is because board members are responsible for coordinating the evaluation by outside experts of a manuscript’s technical aspects and the “importance” or “novelty” of the research it summarizes, i.e., peer review, on which the decision to publish a manuscript is ultimately based. Editors also play a central but underappreciated role in shaping the community of scholars contributing to the discourse in their field. First, by recommending the publication of an article, the editor confers legitimacy not only on the research but also upon the individuals who carried it out [3,4]. Second, editors help choose new editors. In doing so, they confer enhanced status and visibility on a select group of scholars who then benefit from the unique opportunities for professional advancement provided by board membership [5]. Editors are therefore a small but powerful group of “gatekeepers” [2] that select the scientists and ideas shaping the direction of their discipline.

The increased recognition of editor power, along with the results of studies on workforce diversity [6], have heightened concerns about how the composition of editorial boards might influence the peer-review process [7]. For example, it has been suggested that boards whose members are demographically homogenous might converge on a narrow suite of research topics and approaches they consider worthy of publication [3,4]. This narrow vision—and the board structure driving it—could be perpetuated by editors nominating collaborators whose perspectives and backgrounds likely match their own for board service. Indeed, this is among the principal reasons put forward to explain why women remain severely underrepresented on editorial boards across academic fields [5], which has consequences for the selection of referees and other critical aspects of the editorial process [8].

Recent decades have seen the rapid globalization of research in science, technology, engineering, and math (STEM), resulting in greater representation in international journals of authors based in the “Global South” [9,10], i.e., the world’s “developing” or “emerging” economies located primarily in Latin America, Asia, Africa, and the Middle East. Having editorial boards that reflect this increasing geographic diversity of the global scientific community is thought to benefit both journals and disciplines in ways that parallel the benefits resulting from other forms of diversity. In field-based sciences such as ecology or geology, for example, editors based in the region where studies are conducted will be more familiar with the environmental, social, and economic context and constraints under which they were carried out [11]. This could ensure both more rigorous review and a fairer assessment of reviewer criticisms and proposed improvements. Furthermore, scientists trained in different parts of the world can also have very different epistemological orientations. Increasing geographic diversity on an editorial board could therefore broaden the scope of theoretical and methodological approaches a journal publishes. Ultimately, these benefits of internationalization could help to minimize apparent biases in the review, publication, and citation of articles based on an author’s nationality or home country [10,12].

The first systematic efforts to quantify the nationality of STEM editors—often by using the country in which they were based as a proxy for nationality—were carried out in the early 1980s [13,14]. Since then, a small but growing number of studies have observed similar patterns as these early ones did—individual editorial boards tend to be dominated by scholars from or based in the US and the UK [7]. However, prior studies typically compared board composition of journals using data from only a single calendar year, which makes it impossible to evaluate how the community of gatekeepers has changed over time. Furthermore, most of the journals reviewed are from the physical sciences, medical fields, or lab-based biological sciences [4,15]. As a result, almost nothing is known about the geographic diversity of editors in field-based STEM disciplines [16] such as ecology, evolution, and natural resource management (hereafter, environmental biology [EB]).

The term “diversity” is often used colloquially to refer to the representation of different groups in a focal population or workplace. However, one can formally quantify the diversity of a community (e.g., an assemblage of editors) using a suite of indices derived from information theory. While the indices differ in their assumptions and applications, the most commonly used are calculated using 2 types of information: the number of categories found in a sample (i.e., “richness”) and the relative abundance of these categories (i.e., “evenness”). Most studies of editorial board composition to date only report the number of countries represented by editors, i.e., geographic richness. However, diversity indices permit a more nuanced evaluation of community composition. For example, using only richness might lead one to conclude that the geographic representation of editors based in different countries has remained steady over time, when, in fact, 1 country has become numerically dominant. Another advantage of diversity indices is that they can be compared across groups (e.g., journals), even if the groups differ in richness or population size.

We identified all scientists serving from 1985–2014 on the editorial boards of 24 leading journals in EB (S1 Table) and the countries in which they were based during their board tenure. We then calculated the geographic richness and geographic diversity of this editor community and quantified how it has changed over the last 3 decades. Finally, we assessed the geographic distribution of editors at broader geographic and macroeconomic scales by comparing the representation of editors from different World Bank geographic regions and national income categories. Details on data collection and analysis are in S1 Text; data used in this study are archived at the Dryad repository: https://doi.org/10.5061/dryad.6jn86.2 and https://doi.org/10.5061/dryad.mh189 [17,18].

### How geographically diverse is the editorial community?

**Fig 1:**
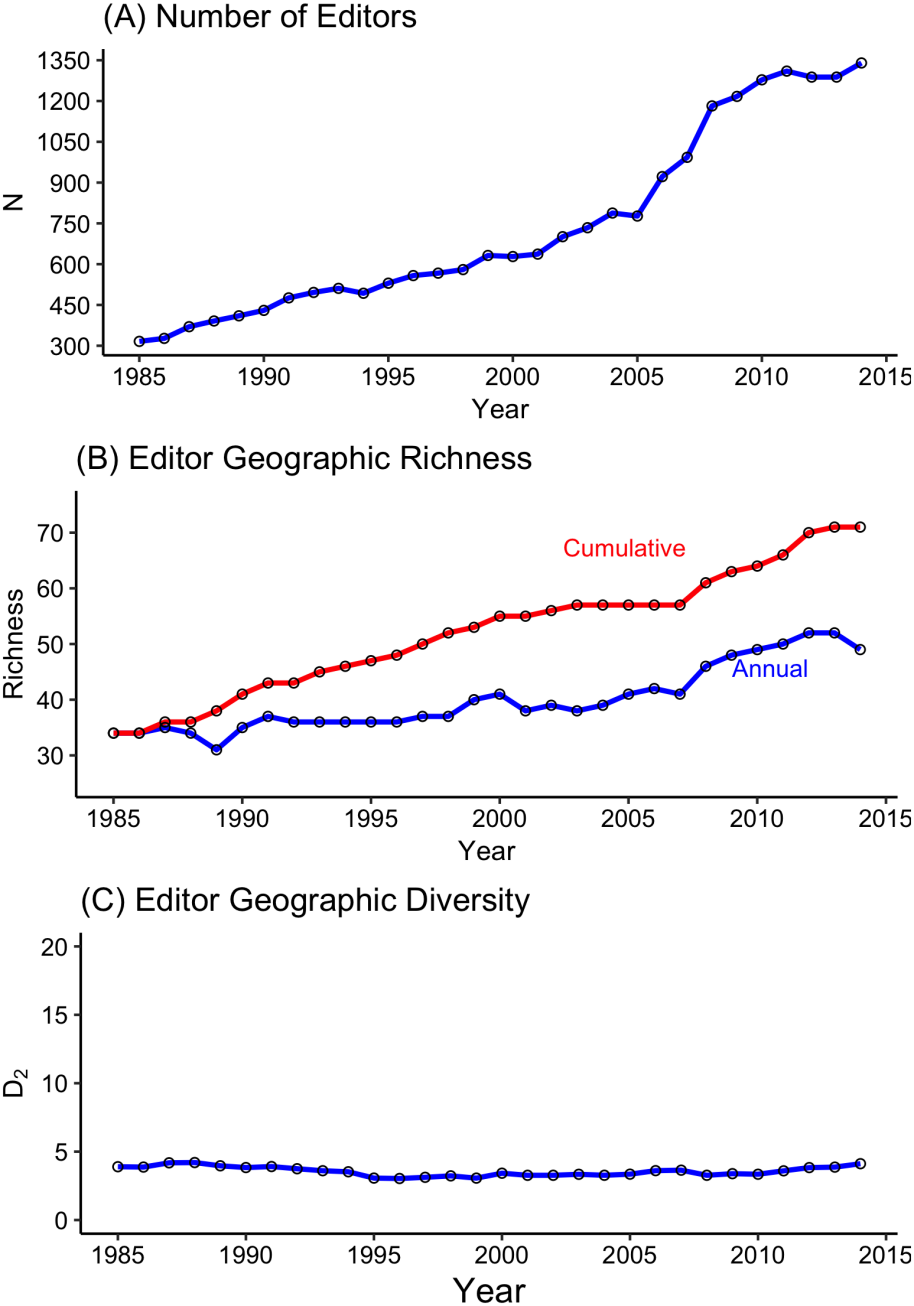
Community composition of editors in environmental biology (1985–2014). (A) Geographic richness: cumulative richness is the total number of countries represented by at least one editor through a given year; annual richness is the total number of countries represented by editors in each year. (B) The total number of unique editors serving each year from 1985 to 2014. (C) The geographic diversity of editors in environmental biology each year from 1985 to 2014. We measured diversity using the reciprocal of *D*_*2*_. Larger values of *D*_*2*_ indicate greater diversity, with the MPD equal to the greatest number of countries represented in any 1 year of the survey (MPD editors = 52). For additional details, see S1 Text. The DOIs for the datasets used in this paper (both archived at Dryad) are https://doi.org/10.5061/dryad.6jn86.2 and https://doi.org/10.5061/dryad.mh189. *D*_*2*,_ Simpson’s index; MPD, maximum potential diversity.

**Fig 2:**
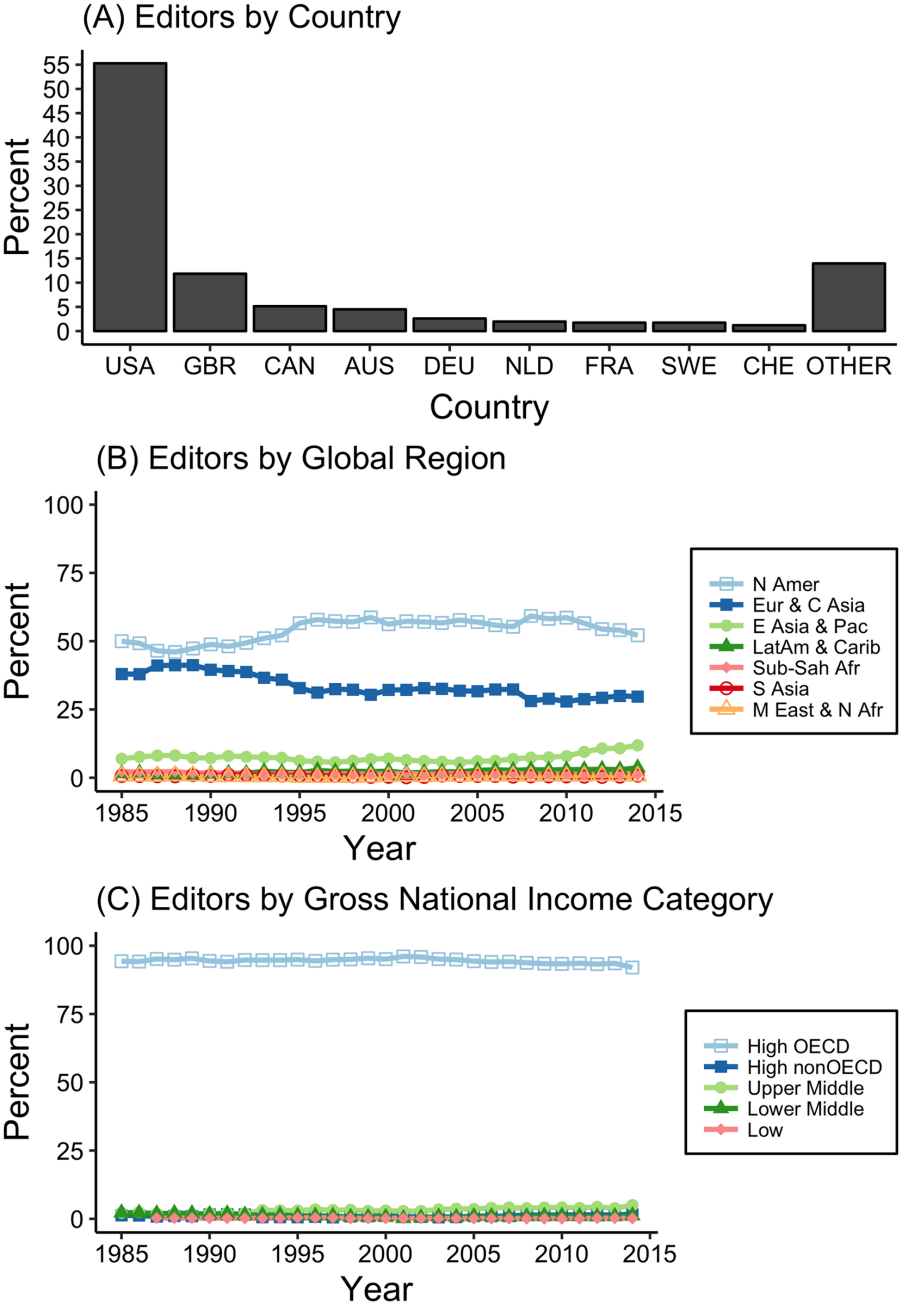
The percentage of environmental biology editors based in different countries, global regions, and World Bank national income categories. (A) Countries. (B) World Bank global regions. (C) World Bank gross national income categories. The DOIs for the datasets used in this paper (both archived at Dryad) are https://doi.org/10.5061/dryad.6jn86.2 and https://doi.org/10.5061/dryad.mh189. AUS, Australia; CAN, Canada; CHE, Switzerland; DEU, Germany; FRA, France; GBR, United Kingdom; NLD, Netherlands; OECD, Organization for Economic Cooperation and Development; SWE, Sweden; USA, United States of America.

Between 1985 and 2014, 3,827 scientists served as editors for our 24 focal journals. The size of the editor community increased steadily over time, with more than 4 times more editors serving in 2014 than in 1985 (Fig 1A). Not surprisingly, this led to an increase in the geographic richness of the editor community—the number of countries represented by editors in 2014 was 44% higher than in 1985 (*N* = 49 versus *N* = 34), and the number of countries to have been represented by at least one editor increased from 34 to 70 (an increase of 109%; Fig 1B). However, scientists based in the US and the UK made up an overwhelming majority of the editor community (55.24% and 11.94%, respectively; Fig 2A). Although there have been modest increases (≤2%) from 1985 to 2014 in the proportion of editors based in 5 other countries (S1 Fig), the continued concentration of editors in a very small number of countries is why the low geographic diversity observed in 1985 has remained unchanged through 2014 (Fig 1C, Table A in S1 Text).

These patterns are echoed when assessing representation at broader geographic or macroeconomic scales. The proportion of editors each year that were based in North America varied from 46%–59%, while 28%–41% were based in Europe/Central Asia (Fig 2B and 2C). The number of editors from the East Asia/Pacific region doubled from 1985 to 2014 (5.6% and 11.9%, respectively; S1 Text Fig B), but most of these were in the high-income countries of Australia, New Zealand, Singapore, and Japan. This concentration of editors in the Global North—the group of economically developed countries with high per capita gross domestic product (GDP) that collectively concentrate most global wealth [19]—was observed at all levels of the gatekeeper hierarchy: 94% of subject and associate editors and a remarkable 98.2% of editors in chief are based in high-income countries or Western Europe (Table 1, S1 Text). In contrast, we found only a fraction of editors have been based in the Global South (Fig 2B and 2C). For example, Brazil, Mexico, and China are represented by fewer editors than Sweden, New Zealand, and the Netherlands (number of editors in 2014: Netherlands = 40, Sweden = 25, New Zealand = 26, China = 22, Brazil = 15, Mexico = 9).

**Table 1:** Percentage of the editorial board members from 24 environmental biology journals based in different (A) World Bank country income categories and (B) global regions. Between 1985 and 2014, there were 3,827 unique editors from 70 countries. The total number of editors in each region and national income category differs due to some editors having moved between 1984 and 2015; similarly, 1 person may serve multiple editorial roles.

**(A) World Bank National Income Category**	**Total Number of Editors**	**Percent of EIC** (*N* = 171)	**Percent of AE** (*N* = 247)	**Percent of SE** (*N* = 3,690)	**Percent of SpE** (*N* = 80)
High-income OECD	3,603	97.66	92.34	93.41	97.50
High-income Non-OECD	50	0.58	1.61	1.30	1.25
Upper-middle income	152	1.75	4.44	4.02	1.25
Lower-middle income	43	0.0	1.61	1.14	0
Low income	5	0.0	0.0	0.14	0
	Total = 3,853				
**(B) Global Region**	**Total Number of Editors**	**Percent of EIC** (*N* = 171)	**Percent of AE** (*N* = 251)	**Percent of SE** (*N* = 3,729)	**Percent of SpE** (*N* = 82)
North America	2,369	50.29	48.41	61.19	67.07
Europe and Central Asia	1,025	45.03	36.11	25.79	23.17
East Asia and Pacific	310	2.34	8.73	7.87	7.32
Latin America and Caribbean	108	0.58	4.37	2.79	1.22
Sub-Saharan Africa	50	1.75	1.59	1.26	1.22
South Asia	23	0.0	0.79	0.62	0
Middle East and North Africa	18	0.0	0.00	0.48	0
	Total = 3,903				

Numbers in parentheses are the number of unique editors in each category. **Abbreviations:** AE, associate editor; EIC, editor in chief; OECD, Organization for Economic Cooperation and Development;; SE, subject editor; SpE, special category editor.

Although several explanations have been put forward to account for this disparity, we believe one of the most common ones—a dearth of capable scientists in the Global South from which to draw [20]—is unlikely to be the cause. The number of scientists in the Global South is increasing dramatically, both in absolute terms and per capita [21], as is their productivity [10,16,22]. Therefore, the number of scientists available to serve each year likely exceeds the number of open editorial positions. While the number of “qualified scientists” is more difficult to quantify, this is also unlikely to be a contributing factor. In 2014 alone, for example, there were over 4,200 scientists based in the Global South who were the lead authors of papers in our focal journals—a pool of scientists 3 times the size of the entire editorial community (S2 Table). Furthermore, 13% of these authors, but only 8% of the editors, were scientists based in middle- and low-income countries, with similar trends for the proportional representation of authors and editors from Africa, the Middle East, Latin America, and the Caribbean (S2 Table). Having said that, we emphasize that it is essential to move beyond proportional representation when thinking of diversity on editorial boards. Why? Because the benefits of diversity continue to accrue as representation increases.

### Why does geographic diversity matter?

Although the increasing geographic richness of editors is a positive development, it is dispiriting that geographic diversity remains unchanged. Unfortunately, it will remain low until a greater proportion of editors are based outside of the US and the UK. But does a lack of geographic representation—be it at the national, regional, or macroeconomic level—have consequences for the process of evaluating manuscripts that could ultimately limit the scope and direction of research in EB? Put bluntly, do editors and reviewers from high-income regions like the US or the UK have biases—implicit or otherwise—that affect how they evaluate submissions from scientists based in the Global South? Although 1 journal in our survey found no evidence that reviewer or author nationality influences the likelihood manuscripts are accepted [23,24], this contrasts sharply with the results of prior studies in other STEM fields [25]. There is also compelling evidence that the region in which authors are based affects where their papers are ultimately published and how much they are cited [10,26,27]. In light of these results and the ample data on how gender and ethnic background influence other aspects of academic evaluation [28], we recommend that editors in chief work to increase the geographic representation on their boards, make editorial board members and referees aware of how biases based on author nationality can affect their editorial judgement, and conduct internal analyses of the potential factors influencing manuscript fate.

Internationalizing editorial boards can also have positive impacts for journals in addition to mitigating possible implicit biases. First, scientists who presume their work will not be judged fairly because of their nationality or where they are based (i.e., the “biased author effect” [29]) may be more likely to submit their manuscripts to journals that have editors representing their region. This both increases the number and scope of submissions a journal receives and the size and expertise of its reviewer pool. Second, a globally diverse editorial board can serve as an important signal of journal quality and connote prestige [29], especially to those tasked with evaluating individual, institutional, or national scientific productivity [15]. Third, it can enhance the profile and impact of the journal and articles published (to say nothing of justification for editors to demand more support or resources from their publishers). Finally, capacity building is central to the mission of academic societies. By providing editorial opportunities to scholars from emerging scientific regions, society journals can play a pivotal role in achieving this goal.

### Geographic diversity: Identifying disparities and setting goals

**Fig 3:**
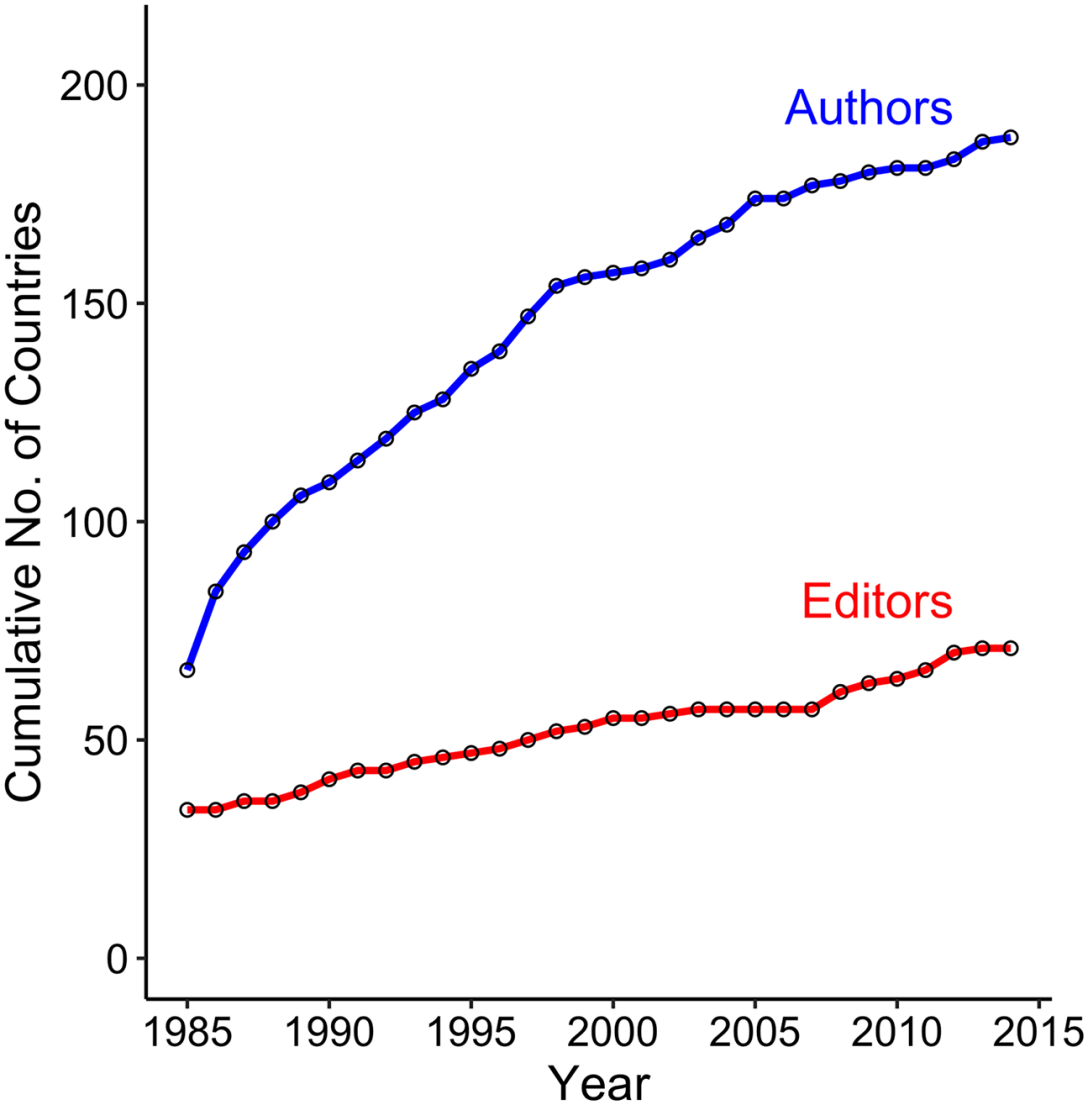
Cumulative geographic richness of editors and authors in environmental biology (1985–2014). Rarefaction curves were generated using data on the editorial board membership of 24 environmental biology journals (S1 Table) and the institutional addresses of authors publishing in those journals (*N* = 113,256 publications; S1 Text). The DOIs for the datasets used in this paper (both archived at Dryad) are https://doi.org/10.5061/dryad.6jn86.2 and https://doi.org/10.5061/dryad.mh189.

Decades of research have highlighted the positive influence of diversity on scientific research teams [30]. Although we recognize editorial boards do not operate in precisely the same way as workplace teams, we believe that increases in their geographic diversity can similarly enhance the creativity and impact of scholarship published in scientific journals. We reiterate prior calls [16] for journal leadership to, at the very least, strive for editorial boards whose regional distribution of editors mirrors that of authors (Fig 3 and S2 Table, S2 Fig). However, we also encourage complementing these efforts by including editors based on criteria such as where a journal’s authors work [11,22] and where their expertise is needed [31,32]. Because the size of editorial boards is typically smaller than the number of countries meeting these criteria, we suggest editors attempt to recruit from less-represented countries within a focal region as opportunities arise.

Regardless of the criteria used to identify areas from which to increase representation, however, efforts must be led by specific plans and timetables to provide both guidance to editors and hold them accountable for their commitments [33]. Whether such plans underlie the geographic diversity we observed on a few of the editorial boards we reviewed is unknown (S3–S6 Figs). Nevertheless, these examples of journals with geographically widespread editors further undermine the frequent argument that it is challenging to find and recruit board members from the Global South with the requisite academic background, editorial experience, and time to serve. We believe that recruiting these editors is the ethical duty of a journal’s leadership, especially given the impact their presence on the board can have on the global scientific community and the diffusion of the knowledge they create in the service of society. Where to find them? We humbly suggest their large and geographically diverse pool of authors (Fig 3, S2 Fig) is an ideal place to start.

## Supporting information

S1 Fig Percent change in the proportion of total editors from different countries from 1985 to 2014. Only countries with changes ±1% are shown. All countries are classified as “High Income: OECD countries” by the World Bank except for China (blue bar), which is in the “Upper Middle Income” category. Abbreviations: GBR: Great Britain, NOR: Norway, CAN: Canada, USA: United States of America, CHN: China, NLD: Netherlands, FRA: France, AUS: Australia.
(TIF)

S2 Fig Proportion of the 1^st^ authors of papers published in N = 24 environmental biology journals in 2014 that were based in different countries. Only the top 15 countries are shown (N = 4266 1^st^ authors total). We also show the proportion of editors serving in 2014 that were based in those countries (red bars). Numbers above the bars indicate the overall ranking of Authors in 2014 and Editors in 2014 (identical numbers indicate ties). Abbreviations: USA: United States of America, AUS: Australia, CAN: Canada, DEU: Germany, CHN: China, FRA: France, NLD: Netherlands, CHE = Chile, ESP = Spain, SWE: Sweden, BRA: Brazil, FIN: Finland, Japan: JPN, MEX: Mexico, ITA: Italy.
(TIF)

S3 Fig Geographic richness of editorial boards in environmental biology (1985–2014). The Geographic Richness (i.e., number of countries represented) of N = 24 environmental biology editorial boards from 1985–2014.
(TIF)

S4 Fig Geographic diversity of editorial boards in environmental biology (1985–2014). Geographic Diversity, calculated as the inverse of Simpson’s Index, D2, of N = 24 environmental biology editorial boards from 1985–2014.
(TIF)

S5 Fig Representation of different global regions on editorial boards in environmental biology (1985–2014). The percentage of editors for each of N = 24 environmental biology journals that are based different global regions (1985–2014).
(TIF)

S6 Fig Representation of national income categories on N = 24 editorial boards (1985–2014). The percentage of editors for each of N = 24 environmental biology journals that are based in countries belonging to different World Bank National Income categories (1985–2014).
(TIF)

S1 Table Focal environmental biology journals. We used N = 24 environmental biology journals in our survey of international representation on editorial boards between 1985–2014.
(DOCX)

S2 Table 2014 editors and authors. The proportion of editors for and first-authors of articles in N = 24 environmental biology journals that are based in different (A) Global Regions and (B) National Income Categories.
(DOCX)

S1 Text Data collection methods, analyses, and results. The study as based on the 1985–2014 Editorial Boards of N = 24 environmental biology journals.
(DOCX)

## Abbreviations

EB: environmental biology
GDP: gross domestic product
STEM: science, technology, engineering, and math
