# Supplementary figures and images for: A persistent lack of international representation on editorial boards in environmental biology
Source: PLoS Biol. 2017 Dec 12;15(12):e2002760. doi: 10.1371/journal.pbio.2002760 (PMC5726619; doi:10.1371/journal.pbio.2002760)

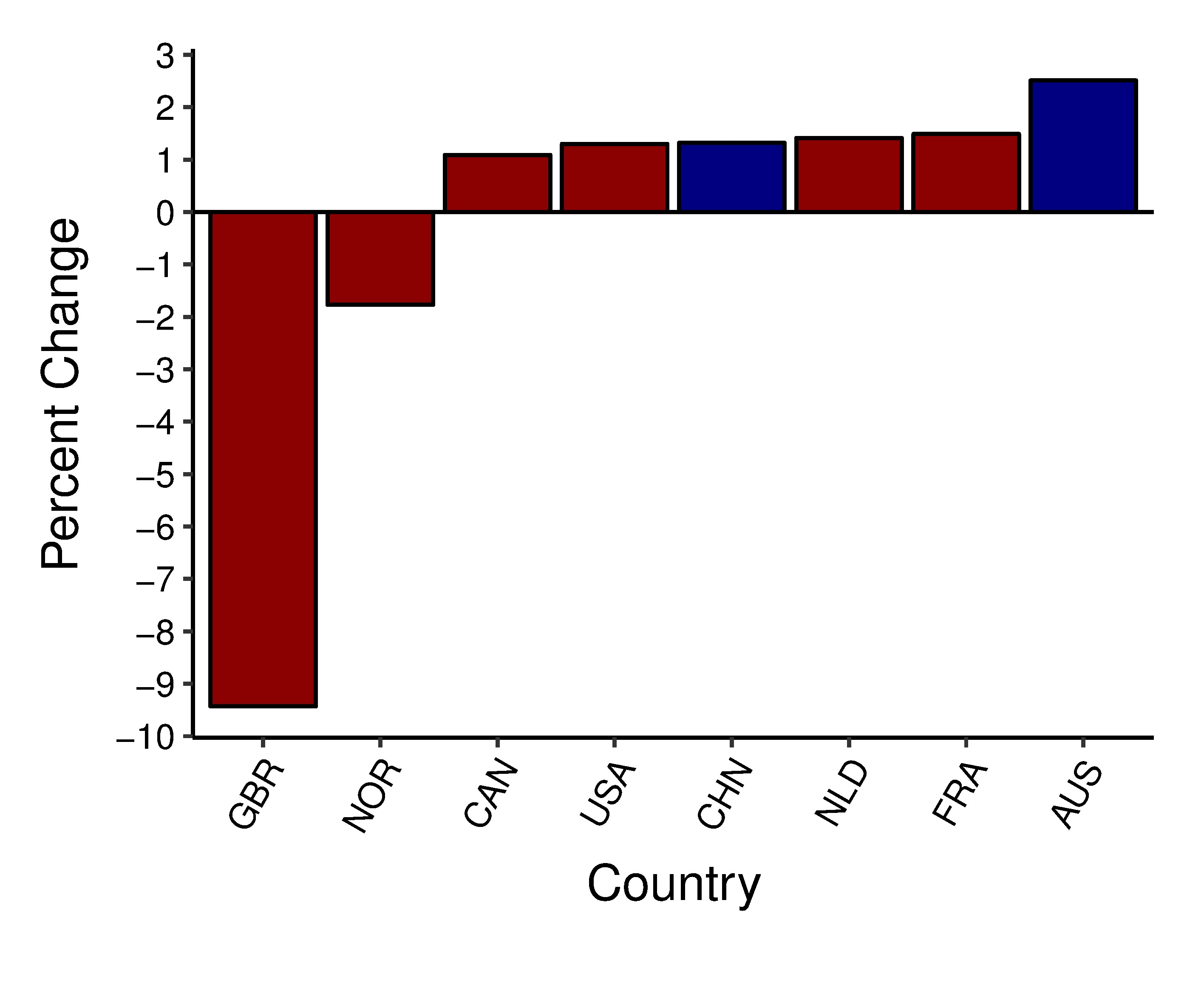

Supplement: S1 Fig — Only countries with changes ±1% are shown. All countries are classified as “High Income: OECD countries” by the World Bank except for China (blue bar), which is in the “Upper Middle Income” category. Abbreviations: GBR: Great Britain, NOR: Norway, CAN: Canada, USA: United States of America, CHN: China, NLD: Netherlands, FRA: France, AUS: Australia. (TIF) [file pbio.2002760.s001.tif]

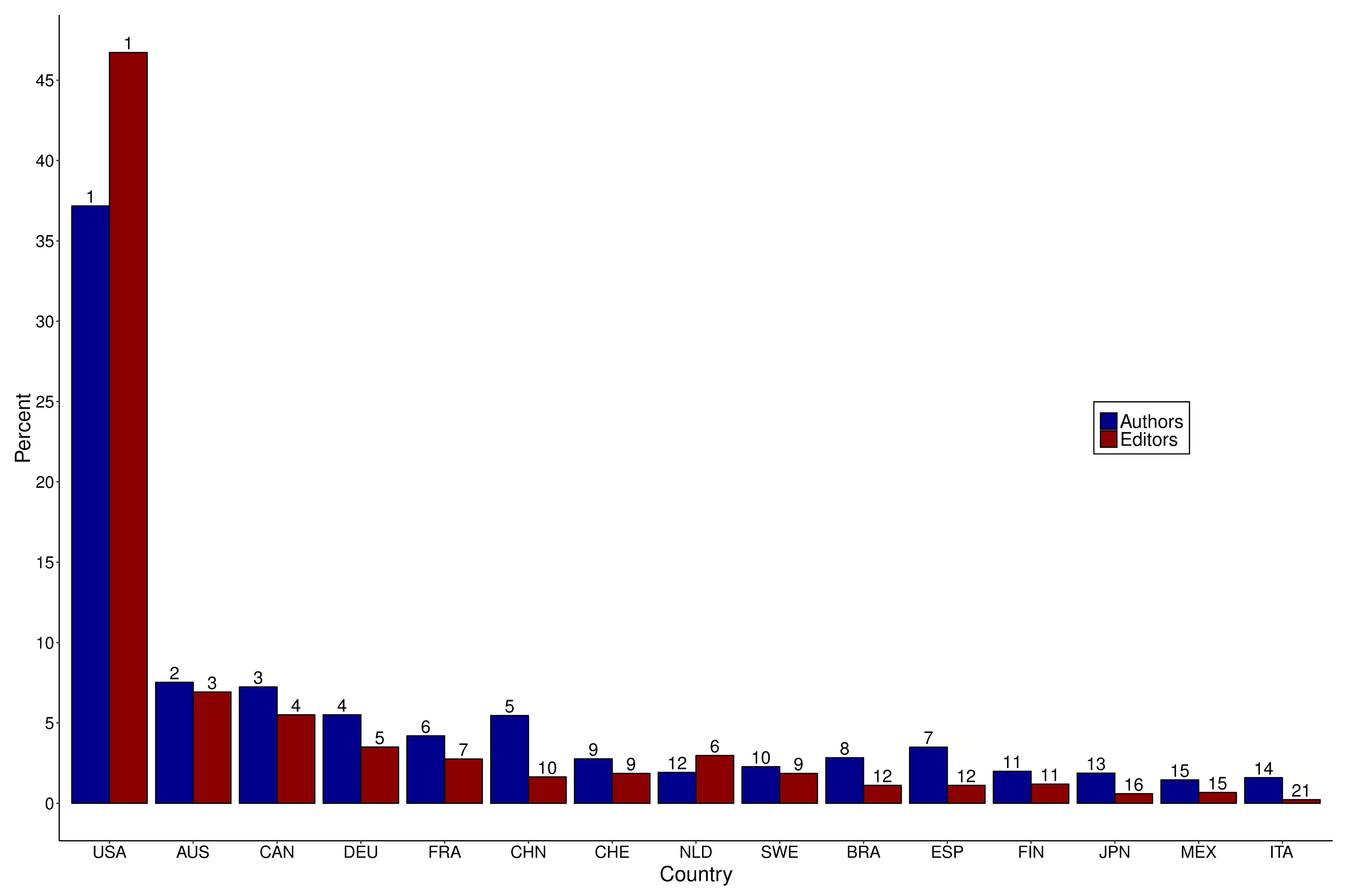

Supplement: S2 Fig — Only the top 15 countries are shown (N = 4266 1st authors total). We also show the proportion of editors serving in 2014 that were based in those countries (red bars). Numbers above the bars indicate the overall ranking of Authors in 2014 and Editors in 2014 (identical numbers indicate ties). Abbreviations: USA: United States of America, AUS: Australia, CAN: Canada, DEU: Germany, CHN: China, FRA: France, NLD: Netherlands, CHE = Chile, ESP = Spain, SWE: Sweden, BRA: Brazil, FIN: Finland, Japan: JPN, MEX: Mexico, ITA: Italy. (TIF) [file pbio.2002760.s002.tif]

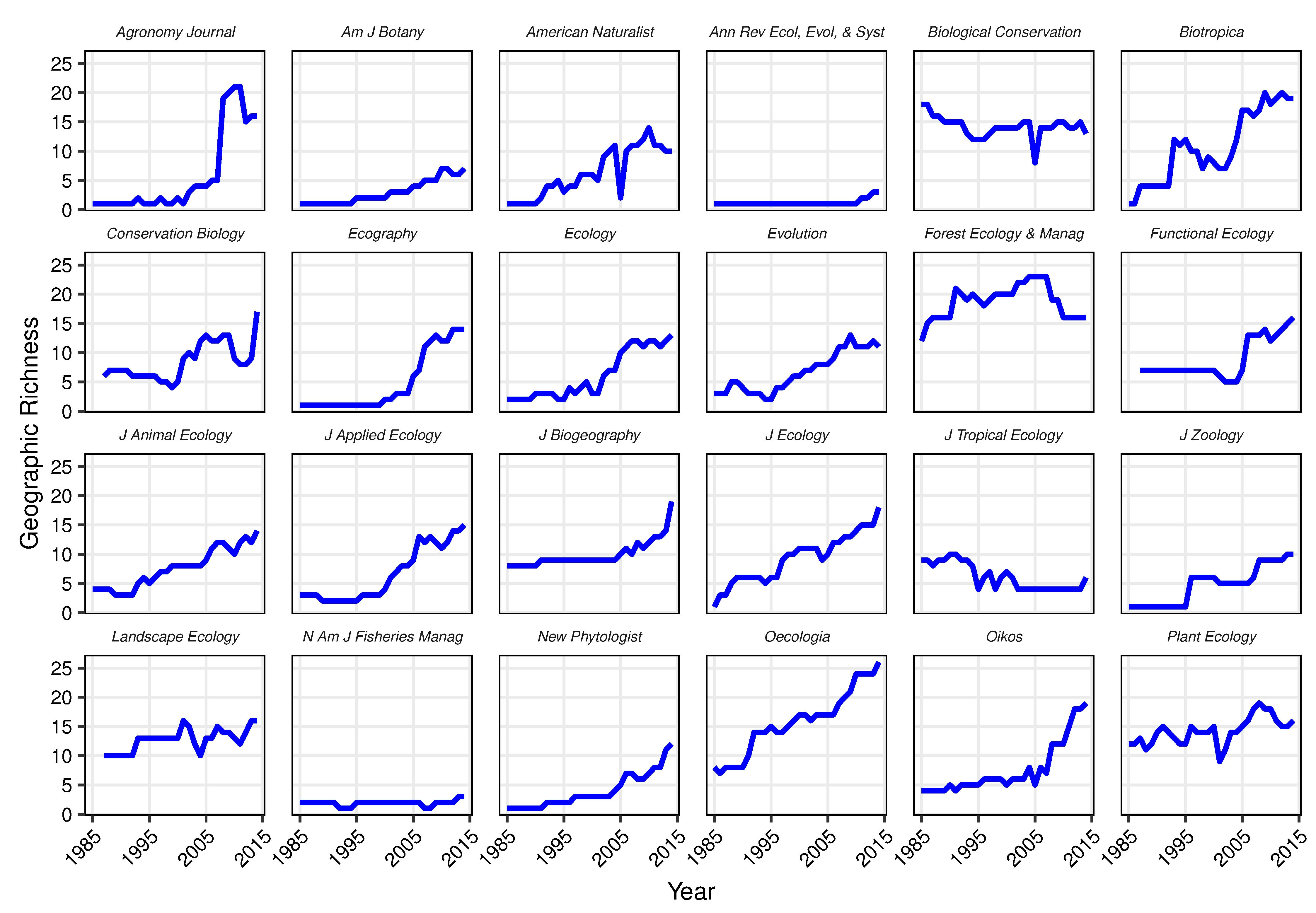

Supplement: S3 Fig — The Geographic Richness (i.e., number of countries represented) of N = 24 environmental biology editorial boards from 1985–2014. (TIF) [file pbio.2002760.s003.tif]

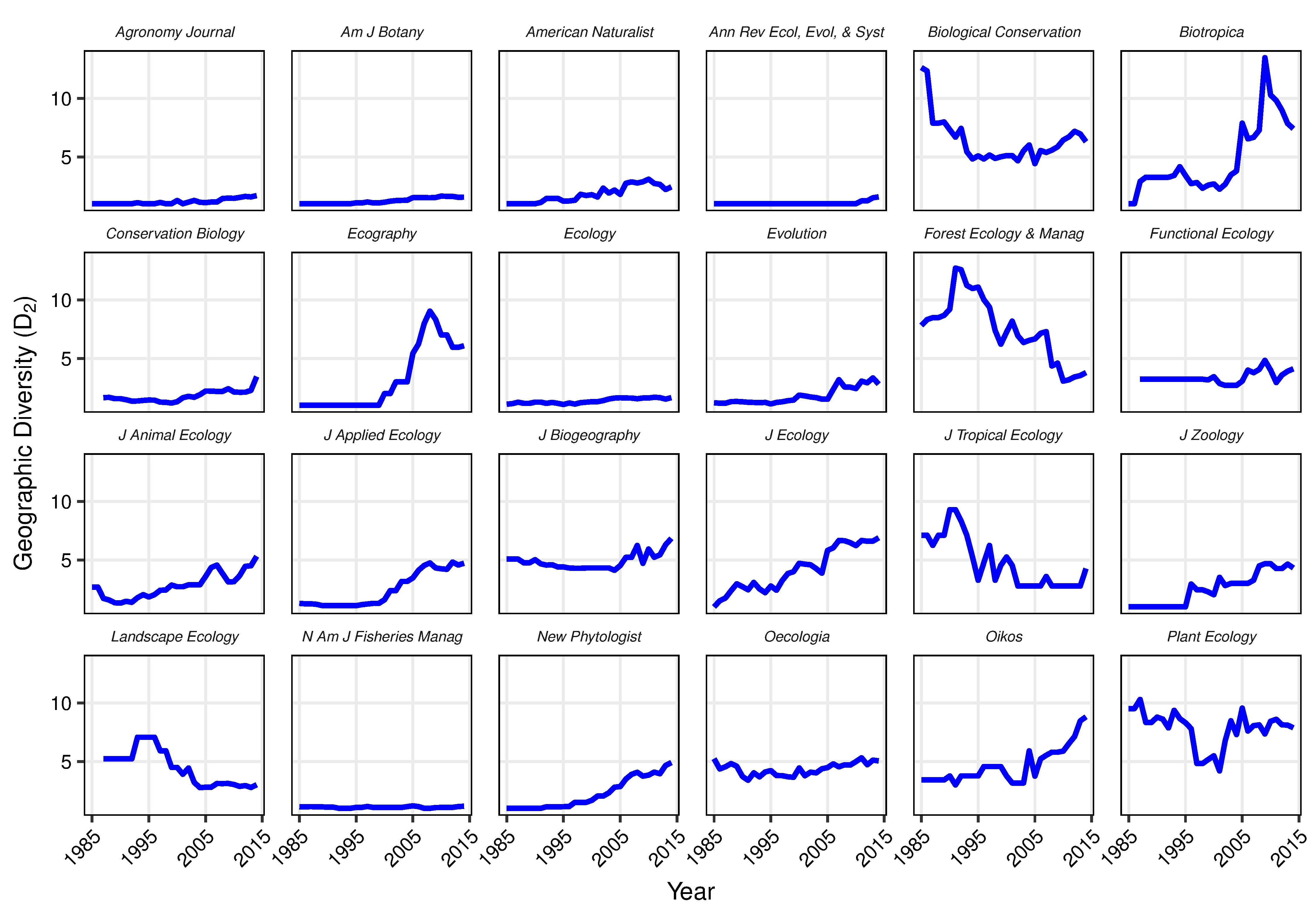

Supplement: S4 Fig — Geographic Diversity, calculated as the inverse of Simpson’s Index, D2, of N = 24 environmental biology editorial boards from 1985–2014. (TIF) [file pbio.2002760.s004.tif]

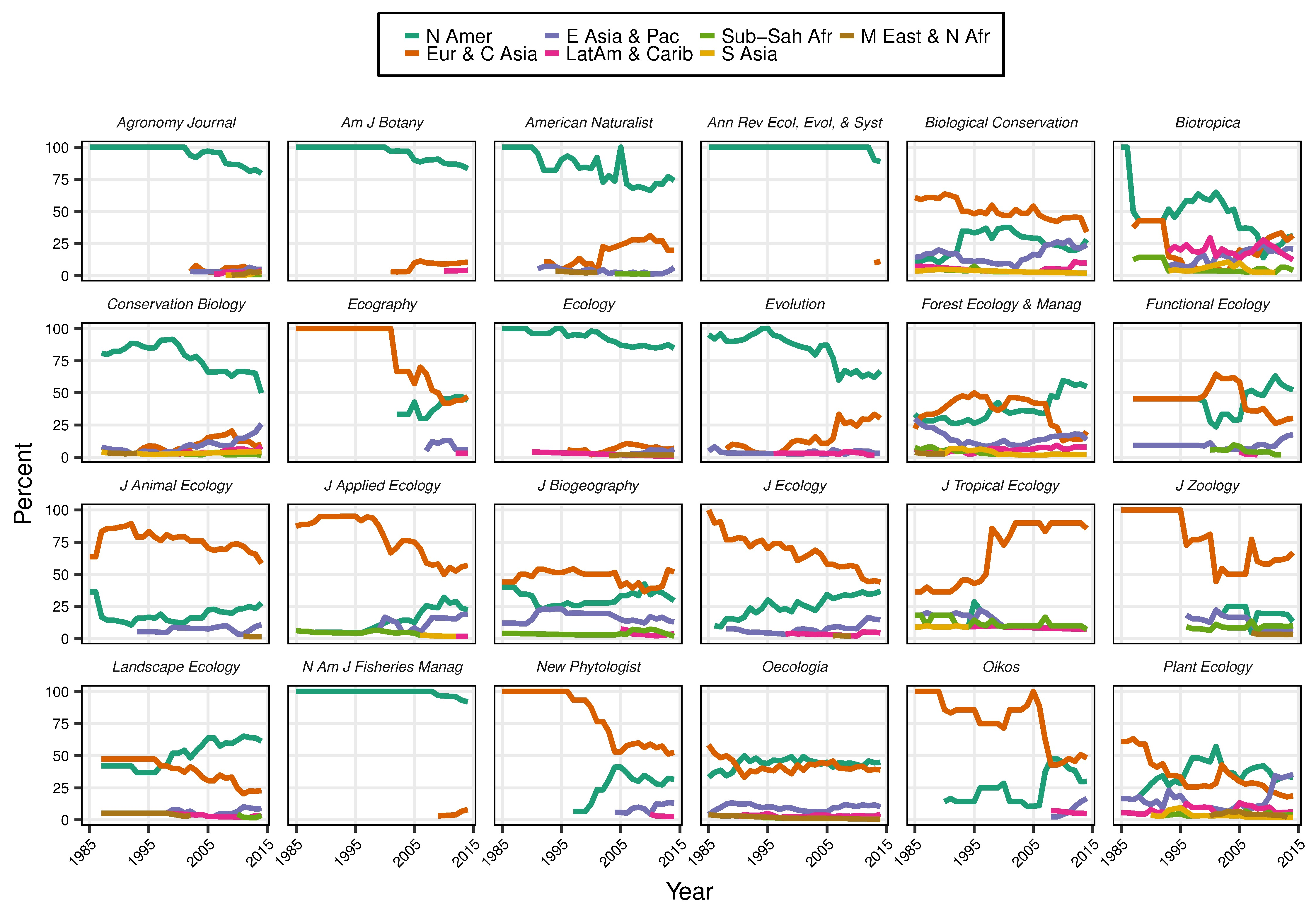

Supplement: S5 Fig — The percentage of editors for each of N = 24 environmental biology journals that are based different global regions (1985–2014). (TIF) [file pbio.2002760.s005.tif]

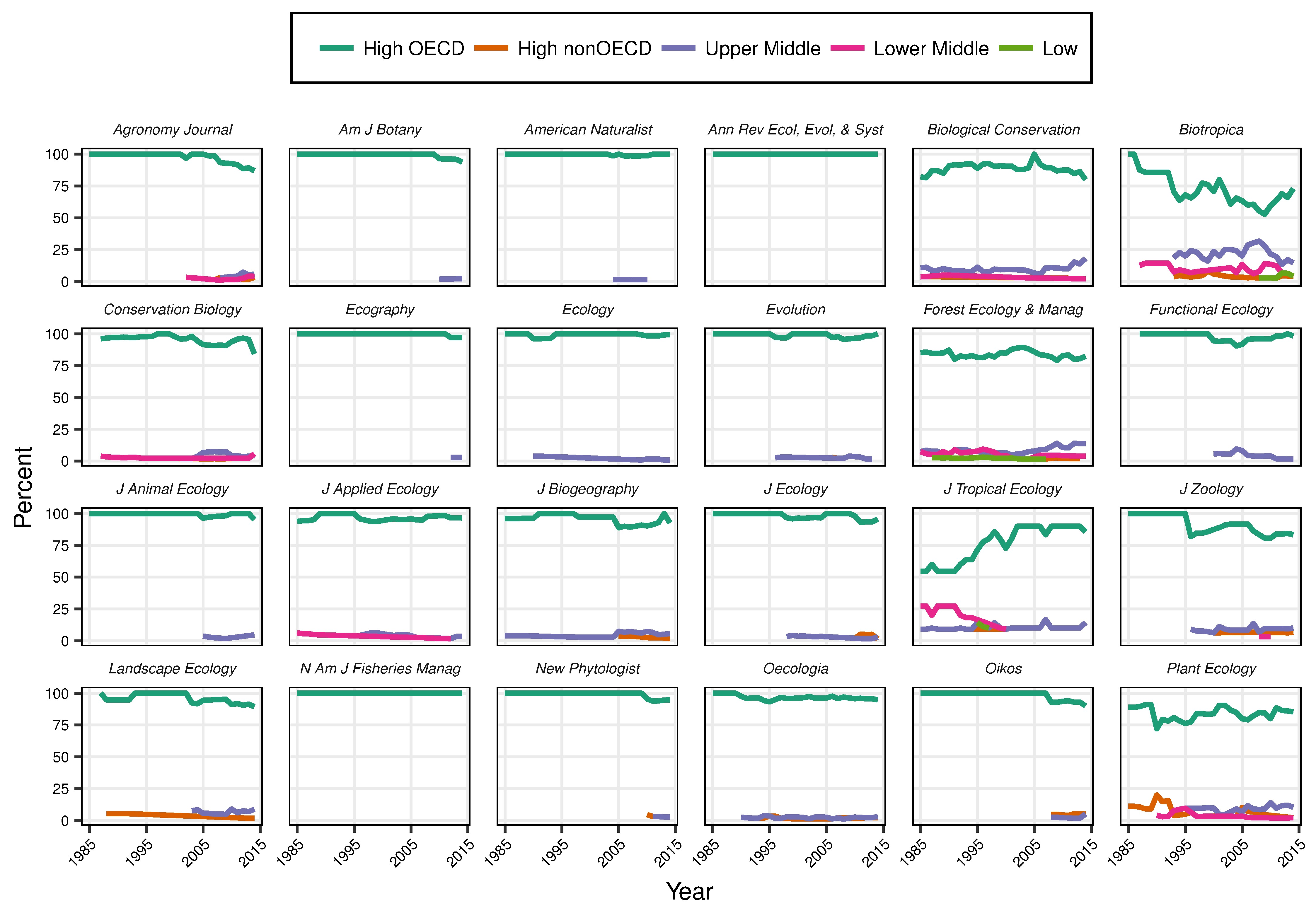

Supplement: S6 Fig — The percentage of editors for each of N = 24 environmental biology journals that are based in countries belonging to different World Bank National Income categories (1985–2014). (TIF) [file pbio.2002760.s006.tif]
