# Supplementary material for: A persistent lack of international representation on editorial boards in environmental biology
Source: PLoS Biol. 2017 Dec 12;15(12):e2002760. doi: 10.1371/journal.pbio.2002760 (PMC5726619; doi:10.1371/journal.pbio.2002760)
Supplement: S1 Table — We used N = 24 environmental biology journals in our survey of international representation on editorial boards between 1985–2014. (DOCX) [file pbio.2002760.s007.docx]

| **Journal** | **Editors**  **1985** | **Countries**  **1985** | **Editors**  **2014** | **Countries**  **2014** | **Total Editors**  **1985-2014** | **Total Countries**  **1985-2014** |
| --- | --- | --- | --- | --- | --- | --- |
| *Agronomy Journal* | 43 | 1 | 127 | 16 | 525 | 24 |
| *American Journal of Botany* | 6 | 1 | 48 | 7 | 116 | 8 |
| *American Naturalist* | 5 | 1 | 66 | 10 | 290 | 18 |
| *Annual Review of Ecology, Evolution, & Systematics^1^* | 8 | 1 | 9 | 3 | 42 | 3 |
| *Biological Conservation* | 28 | 18 | 49 | 13 | 135 | 24 |
| *Biotropica* | 3 | 1 | 48 | 19 | 181 | 36 |
| *Conservation Biology* | 26^4^ | 6^4^ | 70 | 17 | 219 | 23 |
| *Ecography^2^* | 5 | 1 | 34 | 14 | 58 | 16 |
| *Ecology* | 23 | 2 | 130 | 13 | 423 | 18 |
| *Evolution* | 16 | 3 | 63 | 11 | 417 | 20 |
| *Forest Ecology and Management* | 27 | 12 | 51 | 16 | 170 | 34 |
| *Functional Ecology* | 22^4^ | 7^4^ | 63 | 16 | 137 | 20 |
| *Journal of Animal Ecology* | 11 | 4 | 65 | 14 | 135 | 18 |
| *Journal of Applied Ecology* | 16 | 3 | 58 | 15 | 146 | 20 |
| *Journal of Biogeography* | 25 | 8 | 54 | 19 | 123 | 20 |
| *Journal of Ecology* | 10 | 1 | 68 | 18 | 145 | 23 |
| *Journal of Tropical Ecology* | 11 | 9 | 14 | 6 | 36 | 20 |
| *Journal of Zoology* | 1 | 1 | 29 | 10 | 81 | 14 |
| *Landscape Ecology* | 19^4^ | 10^4^ | 57 | 16 | 156 | 26 |
| *New Phytologist* | 10 | 1 | 38 | 12 | 68 | 13 |
| *N. Am. J. of Fisheries Management* | 16 | 2 | 25 | 3 | 252 | 4 |
| *Oecologia* | 24 | 8 | 136 | 26 | 314 | 30 |
| *Oikos* | 12 | 4 | 60 | 19 | 122 | 20 |
| *Plant Ecology*^3^ | 18 | 12 | 47 | 16 | 174 | 32 |

^1^Named *Annual Review of Ecology & Systematics* through 2002. ^2^Named *Holarctic Ecology* through 1991 ^3^Named *Vegetatio* through 1996 ^4^Values for 1987, the first year the journal was published.
