# Supplementary material for: A persistent lack of international representation on editorial boards in environmental biology
Source: PLoS Biol. 2017 Dec 12;15(12):e2002760. doi: 10.1371/journal.pbio.2002760 (PMC5726619; doi:10.1371/journal.pbio.2002760)
Supplement: S2 Table — The proportion of editors for and first-authors of articles in N = 24 environmental biology journals that are based in different (A) Global Regions and (B) National Income Categories. (DOCX) [file pbio.2002760.s008.docx]

**Global Region**

|  | Editors (N) | (%) | Author (N) | (%) |
| --- | --- | --- | --- | --- |
| North America | 699 | 52.16 | 1895 | 44.42 |
| Europe & Central Asia | 397 | 29.63 | 1348 | 31.60 |
| East Asia & Pacific | 159 | 11.87 | 671 | 15.73 |
| Latin America & Caribbean | 49 | 3.66 | 265 | 6.21 |
| Sub-Saharan Africa | 17 | 1.27 | 20 | 0.47 |
| South Asia | 10 | 0.75 | 28 | 0.66 |
| Middle East & North Africa | 9 | 0.67 | 39 | 0.91 |

**Gross National Income Category**

|  | Editors (N) | (%) | Authors (N) | (%) |
| --- | --- | --- | --- | --- |
| High income: OECD | 1232 | 91.94 | 3644 | 85.42 |
| High income: nonOECD | 22 | 1.64 | 464 | 10.88 |
| Upper middle income | 68 | 5.07 | 78 | 1.83 |
| Lower middle income | 16 | 1.19 | 70 | 1.64 |
| Low income | 2 | 0.15 | 10 | 0.23 |
