# Supplementary material for: A persistent lack of international representation on editorial boards in environmental biology
Source: PLoS Biol. 2017 Dec 12;15(12):e2002760. doi: 10.1371/journal.pbio.2002760 (PMC5726619; doi:10.1371/journal.pbio.2002760)
Supplement: S1 Text — The study as based on the 1985–2014 Editorial Boards of N = 24 environmental biology journals. (DOCX) [file pbio.2002760.s009.docx]

**Supporting information for: *A persistent lack of international representation on editorial boards in environmental biology***

Johanna Espin^1,2^, Sebastian Palmas^3^, Farah Carrasco-Rueda^4^, Kristina Riemer^5^, Pablo E. Allen^6^, Nathan Berkebile^4^, Kirsten A. Hecht^4,7^, Kay Kastner-Wilcox^8^, Mauricio M. Núñez-Regueiro^5,*^, Candice Prince^9^, Constanza Rios^4^, Erica Ross^3^, Bhagatveer Sangha^10^, Tia Tyler^9^, Judit Ungvari-Martin^11,7,**^, Mariana Villegas^5^, Tara T. Cataldo^12^, and Emilio M. Bruna^2,5^***

^1^ Dept. of Sociology and Criminology & Law, University of Florida, Gainesville, USA

^2^ Tropical Conservation and Development Program, Center for Latin American Studies, University of Florida, Gainesville, USA

^3^ School of Forest Resources and Conservation, University of Florida, Gainesville, USA ^4^ School of Natural Resources and Environment, University of Florida, Gainesville, USA ^5^ Dept. of Wildlife Ecology & Conservation, University of Florida, Gainesville, USA

^6^ Entomology and Nematology Dept., University of Florida, Gainesville, USA

^7^ Florida Museum of Natural History, University of Florida, Gainesville, USA

^8^ Soil and Water Sciences Dept., University of Florida, Gainesville, USA

^9^ Dept. of Environmental Horticulture, University of Florida, Gainesville, USA

^10^ Horticultural Sciences Dept., University of Florida, Gainesville, USA

^11^ Dept. of Biology, University of Florida, Gainesville, USA

^12^ Martson Science Library, University of Florida, Gainesville, USA

***Corresponding author: Phone: (352) 846-0634, email: embruna@ufl.edu

**CONTENTS**

1. Methods…………………………………………………………………………………..3
   1. Data Collection: Editors………………………………………………………...3
   2. Overview of Analyses: Editors……………………………………….…...……7
   3. Metrics of diversity and community composition…………………………….7
   4. Statistical Analyses: Editors……………………………………………………9
   5. Data Collection and Analysis: Authors………………………………………10
2. Results………………………………………………………………………………….12
3. Table A…..……………………………………………………………………………...14
4. Figure A-B………………………………………………………………………………15
5. References……………………………………………………………………………..17

**1. METHODS**

***1a. Data collection: Editors***

Our analyses are based on the 1985-2014 editorial boards of 24 journals (Table S1). We selected these journals because they are considered high-profile and prestigious outlets in which to publish research from a range of environmental and natural resource disciplines. Whenever possible we selected journals published by academic societies with global membership and comparable publisher-owned outlets for similar research (e.g., *Biotropica* and *Journal of Tropical Ecology*, *Conservation Biology* and *Biological Conservation*). We chose 1985 as a starting point because we wanted to determine if there had been changes in the composition of editorial boards of high-profile disciplinary journals after the emergence of new centers of scientific productivity in Latin America and Asia [1,2]. This meant excluding several high-profile journals because they only began publishing in the past decade (e.g., *Ecology Letters, Molecular Ecology*). We did, however, include three journals that were first published in 1987: *Conservation Biology*, *Functional Ecology,* and *Landscape Ecology* (Table S1).

Using the first issue of the journal published in each calendar year, we recorded the names of all editorial board members, their editorial positions, and the country in which they were based. The 1985-2013 data from 10 of these journals were collected by Cho et al. [3] and archived at the Dryad Digital Repository [4]; we were able to collect the 1986-1989 data for *Journal of Tropical Ecology* missing in Cho et al. for the analyses presented here and include these data in the archived dataset accompanying this paper [5]. After consolidating the two datasets, we disambiguated all author names and assigned each editor a unique identification number.

Journals often have different titles for positions with similar responsibilities; these titles can change over time and new positions are frequently created or eliminated. We therefore used the same definitions as Cho et al. [3] to assign editorial board members to one of four categories based on their primary responsibilities. These categories were:

1. ***Editor-in-Chief (EIC).*** The EIC oversees the journal and is ultimately responsible for editorial policy, standards, and practices, including nominating or appointing new Editorial board members. Some journals have co-Editors-in-Chief (e.g., *North American Journal of Fisheries Management*, *Oecologia*).
2. ***Associate Editors (AE).*** AEs assist the EIC with their responsibilities and often take the lead on some aspects of journal administration. Some AEs oversee all submissions in a specific subject area or about a geographic region. Not all journals have AEs, and some had AEs for only a subset of the survey period.
3. ***Subject Editors (SE).*** SEs oversee manuscript review. SEs for some journals make final decisions on manuscripts after receiving reviewer feedback (e.g., *Ecology*) while SEs for other journals provide recommendations upon on which a senior editor (i.e., EIC, AE) makes the final decision (e.g., *Biotropica, J. Ecology*). They also provide feedback on journal policy and administration. SEs are sometimes referred to by other names, including Handling Editors, the Board of Editors (e.g., *Ecology, Biological Conservation*) and the Editorial Committee (e.g., *Annual Review of Ecology, Evolution, and Systematic, American Journal of Botany*). In addition, two journals used the title of “Associate Editor” for Board members with SE responsibilities (i.e., *American Journal of Botany*, *North American Journal of Fisheries Management*); they were considered SEs in our analyses.
4. ***Special Editors (SpE):*** Special Editors include editors tasked with soliciting papers for special article categories, organizing special sections or volumes, reviewing data archives or computer code, or coordinating reviews of recently published books. Examples of special Editors include those responsible for the “Biological Flora” section of the *Journal of Ecology*, editors for *Ecology’s* “Concept Section”, “Data Archive”, “Special Features”, and “Invited Papers”, the Editors of “Natural History Miscellany” for the *American Naturalist*, and “Commentary” Editors for *Biotropica*. For many journals the Special Editors also serve as the Subject Editors of “standard” manuscript submissions.

We standardized the countries in which editor institutions were based by converting them to their respective ISO 3166-1 alpha-3 codes (ISO 2016). Note that we follow Stocks *et al.* [6] and count editors based in territories or overseas departments separately from those in the sovereign state (e.g., Editors based in Puerto Rico or French Guiana are counted separately from those in, respectively, the USA and France). In cases where the name of the country changed between 1985 and 2014 (e.g., Czechoslovakia, German Democratic Republic) we used the contemporary name for the country where the editor’s home institution was based (e.g., an editor based in Yugoslavia before 1993 would be assigned to Bosnia and Herzegovina, Croatia, Macedonia, Montenegro, Serbia, or Slovenia as appropriate).

We also assigned the country in which each editor was based to its World Bank Global Region and National Income category [7]. The geographic regions are: (1) Europe/Central Asia (2) East Asia/Pacific, (3) Latin America/Caribbean, (4) Sub-Saharan Africa, (5) South Asia, (6) Middle East/North Africa, (7) North America (i.e., Canada and the United States). The National Income categories are: (1) high-income Organization for Economic Cooperation and Development (OECD) member (*per capita* GNI > $12476), (2) high-income non-OECD member (*per capita* GNI > $12476) (3) upper-middle income (*per capita* GNI $4036-$12475), (4) lower-middle income (*per capita* GNI $1026-$4035), (5) low-income (*per capita* GNI < $1025) [7].

Although the country in which an editor is based and the editor’s nationality are frequently conflated [e.g., 8,9], it is important to emphasize that these are not interchangeable [10]. Some studies have avoided this problem by explicitly stating they are using institutional affiliation as a proxy for nationality [e.g., 11], which in some cases may be a reasonable assumption [10]. We make no such assumptions about nationality here – our analyses are explicitly of the country, region, or national economic category in which a scientist is based, irrespective of their citizenship. We do so for several reasons. First, citizenship is not a precondition for serving on editorial boards, and hence it is unlikely to be the reason why scientists are invited (or not) to serve. Second, the national and institutional context in which a scientist is embedded (e.g., availability of financial resources, incentives) likely has a greater impact on their ability to serve as an editor or publish research than their citizenship [6]. Third, some of the skills that make international editors especially important in environmental biology, such as in-depth familiarity with local ecosystems, history, or socio-economic conditions, are also independent of citizenship.

Finally, throughout the text we use the terms “Global North” and “Global South”. The term Global North refers to the group of economically developed countries with high per capita Gross Domestic Product (GDP) that collectively concentrate most global wealth. However, because national development is a product of cultural and political history, not all countries in this classification are located in the Northern Hemisphere (e.g., Australia, New Zealand). The “Global South” comprises the world’s ‘developing’ or ‘emerging’ economies, most of which are in Latin America, Asia, Africa, and the Middle East [12].

***1b. Overview of Analyses: Editors***

Our primary goal was to assess the geographic diversity of the community of scientists serving as editors, not to compare individual journals. We therefore pooled the data from all journals for our analyses. Editors serving on multiple boards in the same year were only counted once. We conducted our analyses using all four editor categories – EIC, AE, SE, and SpE – and use the term ‘editorial board’ to refer to the collection of scientists comprising all four categories. As per Cho et al. [3] we did not include advisors without editorial responsibilities, such as the *American Journal of Botany*’s “Section Representatives” or the “Publication Board” for *Oikos*, nor the staff primarily responsible for the administrative aspects of journal publishing (e.g., production editors, managing editors, editorial assistants).

***1c. Metrics of diversity and community composition***

One can formally quantify the diversity of a group, such as the assemblage of species in a site, using indices derived from information theory [13]. The most commonly used diversity indices are calculated using two types of data: a sample’s “Richness” (i.e., the number of distinct species or categories it contains) and it’s “evenness” (i.e., the relative abundance of each species or category in the sample) [13].

One of the most robust and widely used indices is the reciprocal transformation of Simpson’s Index, *D_2_*, calculated as:

$$D_{2}=\frac{1}{\sum_{i-1}^{R} p_{i}^{2}}$$

where where *R* is the greatest value of richness recorded in any time period sampled between *t*_initial_ and *t*_final_ and *p_i_* is the proportional abundance of type *i* at time *t*. Simpson’s Index has a number of advantages over other common diversity indices (e.g., Shannon’s Index). These include ease of interpretation – when it is expressed as *D_2_*, larger values indicate greater diversity, with maximum potential diversity equal to the greatest value of richness in any one sample year (or site, in the case of spatial comparisons) – and that estimates of diversity for different groups or time intervals are directly comparable, even if they differ in sampling effort or richness [13]. It is important to note that while Simpson’s Diversity will increase with Richness, it is much more sensitive to how equitably individuals are distributed between the different types in a sample (i.e., it is a ‘dominance’ or ‘evenness’ index, *sensu* [13]).

Using our data on board membership, we calculated and report here (1) the Geographic Richness (GR) editors each year from 1985-2014 and (2) the Geographic Diversity of editors each year from 1985-2014 (GD, calculated as *D_2_*). We also generated rarefaction curves to calculate the cumulative Geographic Richness, i.e., the total number of unique countries from 1985 through 2014 in which editors were based.

***1d. Statistical Analyses: Editors***

The organization, visualization, and analysis of data, including the disambiguation of names and assignment of unique identification numbers, was carried out using the R programming language [14] using the tidyr, dplyr, and ggplot2 libraries [15]. All newly collected data have been permanently archived in the Dryad Digital Repository [5]; the version of the code used for the analyses presented in this paper is archived online [16] and is also publicly available for download and improvement [17].

To determine if there were temporal trends in the composition of the editorial community, we calculated the Geographic Richness (GR) and Geographic Diversity (GD) of each year’s community of editors using the vegan library [18]. We then tested for changes in GR and GD over time with linear models fit with Generalized Linear Squares (GLS). We used this approach because it allows testing for and removing the effects of potential temporal autocorrelation resulting from editors serving terms of multiple and consecutive years.

We constructed models in which the dependent variable was the value of each metric in each year and Year and the Number of Editors in a year were included as factors independently or in combination. Preliminary analyses indicated that there was autocorrelation in all response variables, so we included it in all models as an auto-regressive moving average (ARMA) process with *p* = 1 and *q* = 0. We then used Akaike Information Criteria corrected for smaller sample sizes (i.e., AICc) to identify the model whose combination of main effects and interactions provided best fit the data. A significant effect of Year, either alone or in combination with Editor Number, would indicate a change over time in Richness and Diversity. These analyses were carried out using the libraries nlme [19] and MuMIn [20].

Finally, we used χ^2^ tests to compare the number of unique editors (all years combined) based in each World Bank global region and national income category.

***1e. Data collection & Analysis: Authors***

To complement our survey of the community of editors, we also collected data on the country in which the authors of articles published in our focal journals were based. We searched the Thomson-Reuters Web of Science (WOS) Core Collection with the following search string:

*SO=(Agronomy Journal OR American Journal of Botany OR Journal of Applied Ecology OR American Naturalist OR Journal of Biogeography OR Annual Review of Ecology*, OR Journal of Ecology OR Biological Conservation OR Journal of Tropical Ecology OR Biotropica OR Journal of Zoology OR Conservation Biology OR Landscape Ecology OR Ecography OR Holarctic Ecology OR Ecology OR New Phytologist OR Evolution OR North American Journal of Fisheries Management OR Forest Ecology and Management OR Oecologia OR Functional Ecology OR Oikos OR Journal of Animal Ecology OR Plant Ecology OR Vegetatio) AND PY=(X)*

where *X* is each individual year from 1985-2014. We then downloaded the WOS-generated frequency table reporting the countries in which the authors of all items published in the focal journals in each year were based, standardized their home countries using same methods as for editors, and used these data to calculate the annual and cumulative Geographic Richness of article authors from 1985-2014. These WOS-generated summary tables assign authors with institutional addresses in multiple countries equally to each nation, e.g., a paper whose author has a primary address in the USA and a secondary address in Panama results in both Panama and USA being ‘credited’ for that author. This could potentially result in an overestimate of the total number of countries represented by authors.

The WOS-generated analyses of author institutions do not provide the total number of authors from each country, only how many times a country was represented in the articles published in a given year. As such, these data can therefore be used to calculate the Geographic Richness of authors, but not abundance-based metrics such as Geographic Diversity. We therefore complemented the analysis of how the Geographic Richness of authors changed over time by quantifying and comparing the Geographic Diversity of Authors and Editors in 2014. Using the same search string as above, we downloaded the complete WOS records for all articles published in our focal journals in 2014. After disambiguating author names, we selected the first author of each article, identified the country in which their primary institution was located, and assigned each author’s home country to its global region and national gross domestic income category. We then calculated (a) *D_2_* for 1^st^ author home countries (b) the proportion of authors in each global region and (c) the proportion of 1^st^ authors in each national income category. We compared the number of 2014 authors and editors in each region and income category using χ^2^ tests.

**2. RESULTS**

We identified N = 3827 scientists from N = 70 countries that served as editors for our focal journals from 1985 to 2014. Over the course of our survey period the size of the editor community increased almost 420%: from N=316 in 1985 to N=1342 in 2014. The number of countries represented per year increased from N=34 in 1985 to N=49 in 2014.

After accounting for autocorrelation, the increase in Geographic Richness over time was best explained by the number of editors (S1 Text Table A, S1 Text Fig A). In contrast, the best fit for the data on Geographic Diversity was the model that included only the intercept, indicating no increase in diversity over the course of our survey period even after taking into account the increasing number of editors over time (Table A in S1 Text). Finally, there was a significant difference in the frequency of editors representing different national income categories (χ^2^ = 13029, *df* = 4, *p* < 0.0001) and geographic regions (χ^2^ = 8246, *df* = 6, *p* < 0.0001). Editors were overwhelmingly from High-income OECD countries or North America and Europe/Central Asia (Fig 2). The only region to make substantial gains from 1985-2014 was East Asia and the Pacific (5.5% to 11%), though this did not result in greater representation of national income categories (S1 Text Fig B) because most of these editors were based in Australia and New Zealand.

Between 1985-2014 there were 113,256 items published in the N = 24 journals we reviewed. In 1985 the authors of these articles were based in N = 66 countries, but by 2014 the cumulative number of countries represented had almost tripled to N = 189 (Fig 3). In 2014 there were N = 4998 articles published in our focal journals, a 176% increase over the number they published in 1985 (N = 2828). These articles had N = 20,573 unique authors, of which the 4266 lead authors were based in 72 countries (note there were fewer 1^st^ authors than articles because some individuals were lead authors of >1 article). In many cases the ranked order of countries with the most authors were the same as those of the countries represented by the most editors, with several notable exceptions: China had the 5^th^ highest number of authors but was only ranked 10^th^ in editor number, Spain and Brazil were 7^th^ and 8^th^ in author number (respectively) but tied for 12^th^ in Editor number, and Italy was 14th in author number but only 21^st^ in editorial representation (Fig S2).

In 2014 the Geographic Diversity (*D_2_*) of authors was almost 50% higher than that of editors (N = 4266 1^st^ Authors, *D_2_* = 6.15; N = 1344 Editors, *D_2_* = 4.12), and there was a significant difference in the frequency of editors and authors representing different national income categories (χ^2^ = 43.95, *df* = 4, *p* < 0.0001) and geographic regions (χ^2^ = 47.36, *df* = 6, *p* < 0.0001). Authors from middle- and low-income countries made up 13% of the 1^st^ authors publishing in our focal journals in 2014 but only 8% of the Editors, with similar trends for global regions (e.g., Latin America and Africa: 8.3% of authors vs. 5.2% of editors; Table S2). In 2014 there were 31 countries represented by authors but not editors; 75% of these countries were in middle- or low-income countries.

**TABLE A.** Model selection for the effect of Year (model 2), the Total Number of Editors (model 3), both Year and Total Number of Editors (model 4), and Year, Editor Number, and their Interaction (Model 5) on three metrics of editor community composition fit to 30 observations (i.e., total degrees of freedom). All models included an ARMA(1) autocorrelation term. The best-fit model is indicated in bold.

**Geographic Richness**

| Model | dAIC | df | weight |
| --- | --- | --- | --- |
| 1 Intercept | 17.44 | 3 | 0 |
| 2 Year | 11.34 | 4 | 0.003 |
| **3 No. of Editors** | **0** | **4** | **0.75** |
| 4 Year + No. of Editors | 2.76 | 5 | 0.19 |
| 5 Year * No. of Editors | 5.29 | 6 | 0.05 |
|  |  |  |  |

**Geographic Diversity**

| Model | dAIC | df | weight |
| --- | --- | --- | --- |
| **1 Intercept** | **0** | **3** | **0.45** |
| 2 Year | 2.66 | 4 | 0.12 |
| 3 No. of Editors | 2.65 | 4 | 0.12 |
| 4 Year + No. of Editors | 5.17 | 5 | 0.03 |
| 5 Year * No. of Editors | 0.93 | 6 | 0.28 |
|  |  |  |  |

**Fig. A.** Relationship between Geographic Richness and the size of the Editor community (1995-2014, pooled data from N=24 journals).

**Fig. B.** Proportion of editors from N = 24 environmental biology journals based in less represented (A) Global Regions and (B) National Income Categories (1985-2014). Abbreviations: N Amer: North America, Eur & C Asia: Europe & Central Asia, E Asia & Pac: East Asia & Pacific, LatAm and & Carib: Latin America and Caribbean, S Asia: South Asia, M East & N Afr: Middle East & North Africa.

**REFERENCES**

1. Holmgren M, Schnitzer SA. Science on the rise in developing countries. Plos Biol. 2004;2(1):e1. doi: 10.1371/journal.pbio.0020001.

2. Smith MJ, Weinberger C, Bruna EM, Allesina S. The scientific impact of nations: Journal placement and citation performance. PLoS ONE. 2014;9(10):e109195. doi: 10.1371/journal.pone.0109195.

3. Cho AH, Johnson SA, Schuman CE, Adler JM, Gonzalez O, Graves SJ, et al. Women are underrepresented on the editorial boards of journals in environmental biology and natural resource management. PeerJ. 2014;2:e542. doi: 10.7717/peerj.542.

4. Cho AH, Johnson SA, Schuman CE, Adler JM, Gonzalez O, Graves SJ, et al. Data from "Women are underrepresented on the editorial boards of journals in environmental biology and natural resource management (PeerJ)". 2014; Database: Dryad Digital Repository.Available from: <http://doi.org/10.5061/dryad.6jn86>.

5. Espin J, Sebastian Palmas, Carrasco-Rueda F, Riemer K, Allen PE, Berkebile N, et al. Data from: A persistent lack of international representation on editorial boards in environmental biology 2017; Data will be be uploaded to Dryad upon acceptance of the manuscript.

6. Stocks G, Seales L, Paniagua F, Maehr E, Bruna EM. The geographical and institutional distribution of ecological research in the tropics. Biotropica. 2008;40(4):397-404. doi: 10.1111/j.1744-7429.2007.00393.x.

7. The World Bank. World Bank Country and Lending Groups. 2017. Available from: <https://datahelpdesk.worldbank.org/knowledgebase/articles/906519-world-bank-country-and-lending-groups>.

8. Garcia-Carpintero E, Granadino B, Plaza LM. The representation of nationalities on the editorial boards of international journals and the promotion of the scientific output of the same countries. Scientometrics. 2010;84(3):799-811. doi: 10.1007/s11192-010-0199-3.

9. Braun T, Diospatonyi I. Counting the gatekeepers of international science journals a worthwhile science indicator. Current Science. 2005;89(9):1548-51.

10. Bohannon J. Restless minds. Science. 2017;356(6339):690-2. doi: 10.1126/science.356.6339.690.

11. Clark DB. Ecological field studies in the tropics: geographical origin of reports Bulletin of the Ecological Society of America. 1985;66:6-9.

12. Independent Commission on International Development Issues. North-South: a programme for survival: report of the Independent Commission on International Development Issues. Cabridge, MA: MIT Press; 1980.

13. Magurran AE. Measuring biological diversity. Oxford: Wiley-Blackwell; 2004.

14. R Core Team. R: A language and environment for statistical computing. 2016; R Foundation for Statistical Computing, Vienna, Austria.Available from: [https://www.r-project.org/](https://www.R-project.org/)

15. Wickham H, Grolemund G. R for data science. Sebastopol, CA: O’Reilly; 2016.

16. Bruna EM. embruna/Espin_etal_Editors: Espin_etal_PlosBio_Code (Version v1.0.0). Zenodo. http://doi.org/10.5281/zenodo.1067800. 2017.

17. Bruna EM. GitHub repository for code used in Espin et al. A persistent lack of international representation on editorial boards in environmental biology. 2017. Available from: <https://github.com/embruna/Espin_etal_Editors>.

18. Oksanen J, Blanchet FG, Friendly M, Kindt R, Legendre P, McGlinn D, et al. vegan: Community Ecology Package. R package version 2.4-2. 2017; [https://cran.r-project.org/package=vegan](https://CRAN.R-project.org/package=vegan)

19. Pinheiro J, Bates D, DebRoy S, Sarkar D, R Core Team. nlme: Linear and Nonlinear Mixed Effects Models. R package version 3.1-128. 2016; [https://cran.r-project.org/package=nlme](https://CRAN.R-project.org/package=nlme).

20. Bartoń K. MuMIn: Multi-Model Inference. R package version 1.15.6. 2016; [https://cran.r-project.org/package=MuMIn](https://CRAN.R-project.org/package=MuMIn)
